# Bioactivities of a New Pyrrolidine Alkaloid from the Root Barks of *Orixa japonica*

**DOI:** 10.3390/molecules21121665

**Published:** 2016-12-02

**Authors:** Xin Chao Liu, Daowan Lai, Qi Zhi Liu, Ligang Zhou, Qiyong Liu, Zhi Long Liu

**Affiliations:** 1 Department of Entomology, China Agricultural University, Haidian District, Beijing 100193, China; liuxinchao@cau.edu.cn (X.C.L.); lqzwyz@cau.edu.cn (Q.Z.L.); 2 Department of Plant Pathology, China Agricultural University, Haidian District, Beijing 100193, China; dwlai@cau.edu.cn (D.L.); lqzhou@cau.edu.cn (L.Z.); 3 State Key Laboratory of Infectious Disease Prevention and Control, Collaborative Innovation Center of Diagnosis and Treatment of Infectious Diseases, National Institute for Communicable Disease Control and Prevention, Chinese Center for Disease Control and Prevention, Beijing 102206, China; liuqiyong@icdc.cn

**Keywords:** *Orixa japonica*, mosquito, pyrrolidine alkaloid, larvicidal activity, nematicidal activity

## Abstract

A new pyrrolidine alkaloid named (*Z*)-3-(4-hydroxybenzylidene)-4-(4-hydroxyphenyl)-1-methylpyrrolidin-2-one was isolated from the ethanol extract of the root barks of *Orixa japonica.* The structure of the new alkaloid was elucidated on the basis of NMR and MS analysis. The compound exhibited larvicidal activity against the fourth instar larvae of *Aedes aegypti* (LC_50_ = 232.09 μg/mL), *Anopheles sinensis* (LC_50_ = 49.91 μg/mL), and *Culex pipiens pallens* (LC_50_ = 161.10 μg/mL). The new alkaloid also possessed nematicidal activity against *Bursaphelenchus xylophilus* (LC_50_ = 391.50 μg/mL) and *Meloidogynein congnita* (LC_50_ = 134.51 μg/mL). The results indicate that the crude ethanol extract of *O. japonica* root barks and its isolated pyrrolidine alkaloid have potential for development into natural larvicides and nematicides.

## 1. Introduction

Mosquito-borne diseases affect at least 500 million people around the world [1]. Dengue fever, mainly transmitted by the yellow fever mosquito (*Aedes aegypti* L.) and Asian tiger mosquito (*Aedes albopictus* Skuse), cause 50 million new infections and 24,000 deaths every year [2]. Malaria, mostly transmitted by *Anopheles sinensis* Wiedemann, used to be a horrible threat to Chinese people for a long period of time. As was reported by the World Health Organization (WHO) in the year of 2010, there were still 225 million people living under the threat of malaria annually [3]. Wuchereriasis and epidemic encephalitis B, causing significant morbidity and mortality, are primarily vectored by the house mosquito, *Culex pipiens pallens* Coquillett [4]. Currently, the main way to control mosquitoes involves application of synthetic pesticides, organophosphates (e.g., temephos, fenthion, and malathion), and insect growth regulators (e.g., diflubenzuron and methoprene) [5].

Plant-parasitic nematodes are serious worldwide threats to forestry and agriculture because of their wide range of host plants and short biological cycles [6]. The pine wood nematode, *Bursaphelenchus xylophilus* (Steiner and Buhrer) Nickle, causes pine wilt disease by inducing rapid wilting and leads to death of host pines [7]. The southern root-knot nematode, *Meloidogynein congnita* (Kofold and White) Chitwood, is one of the most devastating nematode groups, resulting in decreased quality and quantity in fruits, vegetables, and crops [8]. In the past, methods in integrated management of plant-parasitic nematodes have extensively relied on synthetic fumigant and non-fumigant nematicides [6,9].

However, synthetic larvicides and nematicides have shown some side effects, such as being toxic to humans and other nontarget organisms, causing soil and underground water pollution, disrupting biological control systems, and so on [10]. Therefore, it is urgent to research and develop environmentally acceptable larvicides and nematicides. Plants contain a rich source of bioactive chemicals, since many plants have been reported to possess larvicidal and nematicidal activities [8,11,12,13,14].

During our mass screening for bioactive natural products from Chinese medicinal herbs or wild plants, *Orixa japonica* Thunb. (Rutaceae) showed potential larvicidal and nematicidal activity. *Orixa japonica* is a shrub distributed in China (Anhui, Fujian, Guizhou, Henan, Hubei, Hunan, Jiangsu, Jiangxi, Shaanxi, Sichuan, Yunnan, and Zhejiang provinces), Japan, and Korea [15]. The whole plant has been used for the treatment of cough, rheumatism, malaria, and dysentery [16]. According to the former studies, its extracts or constituents possess pharmacological and biological activities [17,18,19]. Chemical investigations revealed that several quinoline alkaloids (evolitrine, kokusaginine, orixinone, skimmianine, etc.), coumarins (bergapten, imperatorin, and xanthotoxin), and terpenoids (friedelin, limonene, and γ-terpinene) have been isolated and identified from this plant [10,18]. In continuation of our study for other structural types of bioactive compounds from the ethanol extract of *O. japonica* root barks, we used the method of repeated column chromatographic separation to yield a new pyrrolidine alkaloid, (*Z*)-3-(4-hydroxybenzylidene)-4-(4-hydroxyphenyl)-1-methylpyrrolidin-2-one. To our knowledge, this is the first report of a pyrrolidine alkaloid that has been isolated and identified from *O. japonica*. This paper deals with the isolation and structure determination of this compound, as well as its bioactivities against the three species of mosquitoes and the two species of plant-parasitic nematodes.

## 2. Results and Discussion

### 2.1. Isolated Bioactive Compound

**Figure 1:**
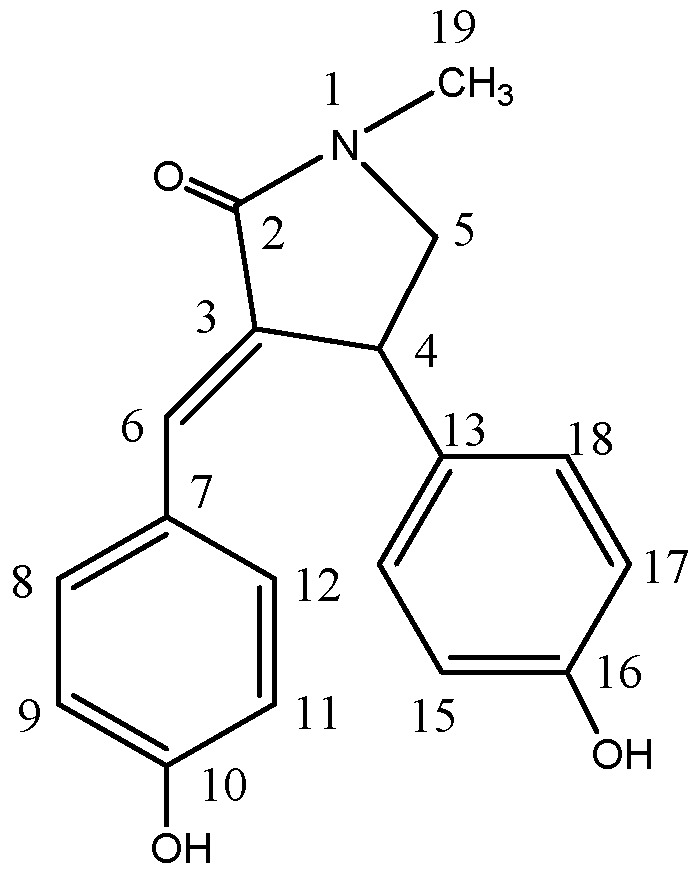
Chemical structure of the pyrrolidine alkaloid isolated from the ethanol extract of *O. japonica* root barks.

Purification of the ethanol extract of *O. japonica* root barks afforded a new pyrrolidine alkaloid (17.9 mg) (Figure 1), the structure of which was elucidated by NMR and MS analysis. NMR and MS spectra are available in supplementary materials.

(*Z*)-3-(4-Hydroxybenzylidene)-4-(4-hydroxyphenyl)-1-methylpyrrolidin-2-one was obtained as white crystal, and displayed a molecular formula of C_18_H_17_NO_3_ ([M − H]^−^, calcd., *m/z* 294.1130; found, *m/z* 294.1135) as determined by high-resolution electrospray ionization mass spectrometry (HRESIMS). The ^1^H- and ^13^C-NMR spectra showed 15 proton and 14 carbon signals (Table 1). According to the HSQC spectrum and a previous report, proton signals at δ_H_ 6.66 (2H, d, *J* = 8.1 Hz), 7.23 (2H, d, *J* = 8.2 Hz), together with their corresponding carbon signals at δ_C_ 114.9 and 131.8, respectively, as well as signals at δ_H_ 6.72 (2H, d, *J* = 8.0 Hz) and 7.05 (2H, d, *J* = 8.0 Hz) with their corresponding carbon signals at δ_C_ 115.3 and 127.7, respectively, suggested the presence of two symmetrical *para*-substituted aromatic protons [20]. Besides, signals at δ_H_ 4.48 (1H, d, *J* = 7.8 Hz), 3.27 (1H, d, *J* = 9.9 Hz), and 3.98 (1H, d, *J* = 9.3 Hz), together with their corresponding carbon signals at δ_C_ 40.33 and 57.0, respectively, and a carbonyl carbon signal at δ_C_ 170.8 revealed a pyrrolidine-2-one ring [21]. Furthermore, the existence of a methyl group at δ_H_ 2.98 (3H, s) was noticeable in the ^1^H-NMR spectrum.

**Figure 2:**
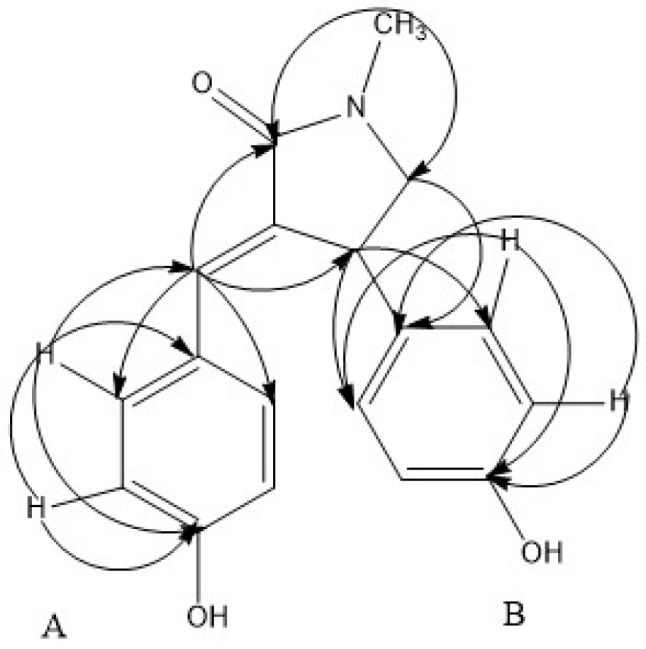
Key HMBC (H→C) correlations of the pyrrolidine alkaloid isolated from the ethanol extract of *O. japonica* root barks.

In the HMBC spectrum, correlations from H-6 (δ_H_ 7.39) to C-8 (δ_C_ 131.8) and C-12 (δ_C_ 131.8), from H-8 (δ_H_ 7.23) to C-6 (δ_C_ 132.2) and C-10 (δ_C_ 158.2), from H-9 (δ_H_ 6.66) to C-7 (δ_C_ 125.9) and C-10 (δ_C_ 158.2), from H-11 (δ_H_ 6.66) to C-7 (δ_C_ 125.9) and C-10 (δ_C_ 158.2), and from H-12 (δ_H_ 7.23) to C-6 (δ_C_ 132.2) and C-10 (δ_C_ 158.2) revealed fragment A (Figure 2). Besides, HMBC correlations from H-14 (δ_H_ 7.05) to C-4 (δ_C_ 40.3) and C-16 (δ_C_ 156.1), from H-15 (δ_H_ 6.72) to C-13 (δ_C_ 133.5) and C-16 (δ_C_ 156.1), from H-17 (δ_H_ 6.72) to C-13 (δ_C_ 133.5) and C-16 (δ_C_ 156.1), and from H-18 (δ_H_ 7.05) to C-14 (δ_C_ 127.7) and C-16 (δ_C_ 156.1) confirmed fragment B (Figure 2). Furthermore, correlations from H-5 (δ_H_ 3.27) to C-2 (δ_C_ 170.8) and from H-19 (δ_H_ 2.98) to C-2 (δ_C_ 170.8) and C-5 (δ_C_ 57.0) proved the existence of fragment C (Figure 2). According to HMBC correlations from H-6 (δ_H_ 7.39) to C-2 (δ_C_ 170.8) and C-4 (δ_C_ 40.3), fragment A was attached to C-3 of fragment C (Figure 2). HMBC correlations from H-4 (δ_H_ 4.48) to C-14 (δ_C_ 127.7) and C-18 (δ_C_ 127.7) revealed fragment B connected with C-4 of fragment C. Thus, the compound was determined as (*Z*)-3-(4-hydroxybenzylidene)-4-(4-hydroxyphenyl)-1-methylpyrrolidin-2-one.

### 2.2. Bioactivities

The isolated pyrrolidine alkaloid was tested for its larvicidal activity against the fourth instar larvae of *Ae. aegypti*, *An. sinensis*, and *C. pipiens pallens*. The fourth instar larvae of *An. sinensis* was the most susceptible with a 24 h LC_50_ value of 49.91 μg/mL (Table 2), while *C. pipiens pallens* and *Ae. aegypti* were 3.2 and 4.7 times less sensitive than *An. sinensis*, respectively. However, such differences in sensitivity to chemicals need to be further investigated. When compared with a positive control, commercial rotenone, the isolated compound was 62–85 times less toxic to the three species of mosquitoes.

The isolated alkaloid also possessed nematicidal activity against juveniles of *Bursaphelenchus xylophilus* and *Meloidogynein congnita* with 72 h LC_50_ values of 391.50 μg/mL and 134.51 μg/mL (Table 3), respectively; *M. incongnita* was 2.9 times more susceptible than *B. xylophilus*. However, this susceptibility difference is not yet clear. In comparison to a positive control, commercial avermectin, the isolated compound was 5000 times less toxic to the two species of nematodes.

On the basis of a literature survey, compounds of the pyrrolidine type have exhibited various biological activities, such as antioxidative, antifungal, and antibacterial activity; antitumor properties; cytotoxicity; and glycosidase inhibitory; and platelet aggregation inhibitory effects [22,23,24]. It is worth mentioning that pyrrolidines have been found to possess larvicidal activity against *Ae. aegypti* at a concentration less than 140 ppm [25]. Pyrrolidines also possess considerable insecticidal activity when evaluated against termite workers [26]. Moreover, pyrrolidine alkaloids have also been found to possess DNA-binding affinity and cytotoxicity [27], which might be the mechanism of action of the new alkaloid against larval mosquitoes and plant-parasitic nematodes. The above findings suggested the new alkaloid with a pyrrolidine ring isolated from the ethanol extract of *O. japonica* has the potential to be developed into an alternative larvicide and nematicide.

## 3. Experimental Section

### 3.1. General

NMR spectra were recorded on Bruker (Billerica, MA, USA) Avance 500 instruments using MeOD as solvents. HRESIMS analysis was performed on an APEX II spectrometer (Bruker Daltonic Inc., Billerica, MA, USA).

### 3.2. Mosquitoes

Eggs of *Ae. aegypti* and *An. sinensis* and egg masses of *C. pipiens pallens* were obtained from the Department of Vector Biology and Control, National Institute for Communicable Disease Control and Prevention, Chinese Center for Disease Control and Prevention. Adult mosquitoes were reared in a cage (60 cm × 30 cm × 30 cm) which was placed in a growth chamber (14:10 h light:dark (L:D), 26–28 °C, 70%–80% RH). Adults were maintained on a 10% glucose solution and were allowed to blood-feed on live mice. Adults of both *Ae. aegypti* and *An. sinensis* deposited on strips of moistened filter paper, which were kept in glass beakers. Paper strips with *Ae. aegypti* eggs were kept wet for 24 h and then dehydrated, while *An. sinensis* eggs need to be kept wet. However, egg masses of *C. pipiens pallens* were oviposited in distilled water directly. Then, the egg masses were transported to a clean, white porcelain basin containing distilled water. Eggs or egg masses were kept in distilled water under the same conditions described above. Mosquito larvae were provided with a mixture of pig liver, fish food, and yeast powder (1:1:1) and were not utilized for experiments until they reached the fourth instar.

### 3.3. Nematodes

Colonies of *B. xylophilus* were maintained on *Botrytis cinerea* cultures. The fungus *B. cinerea* was cultured on potato dextrose agar (PDA) in a growth chamber (25–28 °C in dark). When *B. cinerea* was fully grown on PDA, *B. xylophilus* were collected through the modified Baermann funnel technique, washed with a mixture of 0.1% streptomycin sulfate and 0.002% actinone three times to remove any surface bacterial or fungal contaminants and then inoculated on the plate [28]. The plate was maintained in the growth chamber (25–28 °C in dark) until the fungal mycelium were completely consumed by *B. xylophilus.* Then, *B. xylophilus* were collected, washed thoroughly with sterilized distilled water, and used for bioassays immediately. All the bioassays were performed under laboratory conditions at 25–28 °C.

Eggs of *M. incongnita* were extracted from infected roots of tomato (*Lycopersicone sculentum* Mill.). All the tomato plants were reared in a growth chamber (16:8 h L:D, 25–28 °C, 75%–80% RH). When they reached a five-leaf stage, the tomatoes were used for inoculations. After 42 days, infected tomatoes were uprooted and the roots were washed free of soil with distilled water. Egg masses were hand-picked using sterilized tweezers from infected roots and then placed on a mesh nylon filter (openings 30 μm in diameter) [29]. J2s that passed through the filter were collected daily and used for bioassays immediately.

### 3.4. Plant Material

Fresh root barks of *O. japonica* were collected in Xixiu District, Anshun City, Guizhou Province, China (39.90°N and 116.41°E) in August 2014, and identified by Dr. Liu QR, College of Life Sciences, Beijing Normal University, Beijing. A voucher specimen of *O. japonica* (Rutaceae-*Orixa japonica*-Guizhou-2014-08) was deposited at the museum of the Department of Entomology, China Agricultural University, Beijing, China.

### 3.5. Extraction and Isolation

Root barks of *O. japonica* (20 kg) were air-dried, cut into pieces, and then successively extracted with 40L of different concentrations of ethanol/distilled water mixtures (95%, 75%, 50%, by volume) and distilled water at room temperature for 3 days. The extracts were then filtered, mixed and removed from the solvent under reduced pressure to afford crude extract (200 g). After that, the crude extract was suspended in distilled water (2 L) and then successively partitioned with the same volume of *n*-hexane, chloroform, ethyl acetate, and *n*-butyl alcohol to obtain four solvent fractions (37 g, 69 g, 25 g, 33 g). The *n*-butyl alcohol fraction was subjected to chromatography on a macroporous resin (AB-8, 1000 g) column (85 mm i.d., 850 mm length), eluting with distilled water containing increasing amounts of ethanol (up to 90%, by volume) to yield eight fractions. Fraction 8 (5.3 g) was then subjected to silica gel (Merck 9385) column (50 mm i.d., 500 mm length), eluting with CH_2_Cl_2_–EtOH (100:0–100:50) to afford 25 fractions. Fraction 10 (114 mg) was separated by a silica gel (Merck 9385) column (25 mm i.d., 500 mm length), eluting with CH_2_Cl_2_–EtOH (100:5–100:20) to afford 13 fractions. Fraction 6 (9.2 mg) was further purified by Sephadex LH-20 column (15 mm i.d., 500 mm length) chromatography, eluting with ethanol alone to yield the new pyrrolidine alkaloid (17.9 mg).

### 3.6. Bioactivity Assays

All bioactivity assays were performedunder laboratory conditions at 25–28 °C. The compound was dissolved in ethanol and diluted with tap water. The larvicidal bioassays were as recommended by WHO [30]. Solutions (250 mL) of the tested material at various concentrations were placed in glass beakers, and then 10 larvae were delivered toeach beaker. Each test was composed of six concentrations with five replicates. Commercial rotenone (purchased from Aladdin-Reagent Company, Shanghai, China) served as a positive control and ethanol was used as a negative control. Both treated and control larvae were placed in the growth chamber where mosquitoes were reared. Mortality recordings were taken after 24 h of exposure. Larvae that showed no movements were considered to be dead.

The standard nematode suspensions were prepared by appropriate dilution with sterilized distilled water to get approximately 100 juveniles/mL. Then, each well of 24-well tissue culture plates were added with 500 μL standard juvenile suspension. Numbers of active juveniles in every well were counted under a stereoscope at 10× and 5× before 500 μL stock solution was added to the corresponding well. However, the final concentration of ethanol in each treatment never exceeded 1% (by volume) [31]. Plates were then covered with “Xuan paper” (a high-quality rice paper made for traditional Chinese painting and calligraphy) to avoid evaporation. Each test was composed of five concentrations with three replicates. Commercial avermectin (purchased from Aladdin-Reagent Company, Shanghai, China) was used as a positive control, and distilled water containing ethanol (1%, by volume) served as a negative control. Both treated and control juveniles were placed in a growth chamber at 25–28 °C in dark. Mortality recordings were taken 72 h after treatment. Juveniles that showed no movements when stimulated with a fine needle were considered to be dead.

## 4. Conclusions

A new pyrrolidine alkaloid was obtained from ethanol extract of *O. japonica* root barks, identified as (*Z*)-3-(4-hydroxybenzylidene)-4-(4-hydroxyphenyl)-1-methylpyrrolidin-2-one. It possessed larvicidal activity against fourth instar larvae of *Ae. aegypti*, *An. sinensis*, and *C. pipiens pallens*, as well as nematicidal activity against juveniles of *M. incognita* and *B. xylophilus*. However, further investigations should be conducted to explore its action mechanism and safety issues, so as to develop a fundamental structure with potential bioactivities.

## Figures and Tables

**Table 1:** ^1^H (500 MHz) and ^13^C (125 MHz) NMR data for the isolated compound in MeOD (*J* in Hz).

Position	δ_H_	δ_C_
2		170.8
3		
4	4.48, d, *J* = 7.8 Hz	40.3
5α	3.98, d, *J* = 9.3 Hz	57.0
5β	3.27, d, *J* = 9.9 Hz	
6	7.39, s	132.2
7		125.9
8	7.23, d, *J* = 8.2 Hz	131.8
9	6.66, d, *J* = 8.1 Hz	114.9
10		158.2
11	6.66, d, *J* = 8.1 Hz	114.9
12	7.23, d, *J* = 8.2 Hz	131.8
13		133.5
14	7.05, d, *J* = 8.0 Hz	127.7
15	6.72, d, *J* =8.0 Hz	115.3
16		156.1
17	6.72, d, *J* =8.0 Hz	115.3
18	7.05, d, *J* = 8.0 Hz	127.7
19	2.98, s	29.0

**Table 2:** Larvicidal activity of the isolated pyrrolidine alkaloid against the fourth instar larvae of *Aedes aegypti*, *Anopheles sinensis*, and *Culex pipiens pallens*.

Mosquitoes	Treatment	LC_50_ (μg/mL) (95% CL)	LC_90_ (μg/mL) (95% CL)	Slope ± SD	χ^2^	*p*
*Ae. aegypti*	Compound	232.09 (209.05–254.74)	293.19 (265.30–321.58)	12.63 ± 1.43	8.69	0.0130 *
	Rotenone	3.75 (3.39–4.11)	9.64 (8.64–10.56)	3.22 ± 0.33	18.20	0.0001 *
*An. sinensis*	Compound	49.91 (45.61–54.51)	82.31 (74.12–90.46)	5.90 ± 0.68	11.05	0.0040 *
	Rotenone	0.73 (0.66–0.80)	1.13 (1.02–1.24)	4.00 ± 0.51	10.55	0.0051 *
*C. pipiens pallens*	Compound	161.10 (145.37–177.04)	211.80 (192.01–232.97)	10.78 ± 1.24	9.28	0.0097 *
	Rotenone	1.88 (1.69–2.01)	3.74 (3.38–4.09)	6.90 ± 0.88	15.80	0.0004 *

* Values were significant at the *p* < 0.05 level.

**Table 3:** Nematicidal activity of the isolated pyrrolidine alkaloid against juveniles of *Bursaphelenchus xylophilus* and *Meloidogynein congnita*.

Nematodes	Treatment	LC_50_ (μg/mL) (95% CL)	LC_90_ (μg/mL) (95% CL)	Slope ± SD	χ^2^	*p*
*B. xylophilus*	Compound	391.50 (353.90–429.18)	>500	2.37 ± 0.20	15.15	0.0005 *
	Avermectin	0.071 (0.068–0.075)	0.24 (0.22–0.26)	2.45 ± 0.20	14.76	0.0006 *
*M. incongnita*	Compound	134.51 (121.91–146.24)	>500	1.18 ± 0.12	12.00	0.0025 *
	Avermectin	0.025 (0.023–0.027)	0.13 (0.12–0.14)	2.08 ± 0.16	28.21	0.0000 *

* Values were significant at the *p* < 0.05 level.

## Supplementary Materials

Supplementary materials can be accessed at: http://www.mdpi.com/1420-3049/21/12/1665/s1.
