# Supplementary material for: Bioactivities of a New Pyrrolidine Alkaloid from the Root Barks of Orixa japonica
Source: Molecules. 2016 Dec 2;21(12):1665. doi: 10.3390/molecules21121665 (PMC6273699; doi:10.3390/molecules21121665)
Supplement: Supplementary file 1 [file molecules-21-01665-s001.pdf]

Supplementary Materials: Bioactivities of a New Pyrrolidine Alkaloid from the Root Barks of *Orixa japonica*

Xin Chao Liu, Daowan Lai, Qi Zhi Liu, Ligang Zhou, Qiyong Liu and Zhi Long Liu

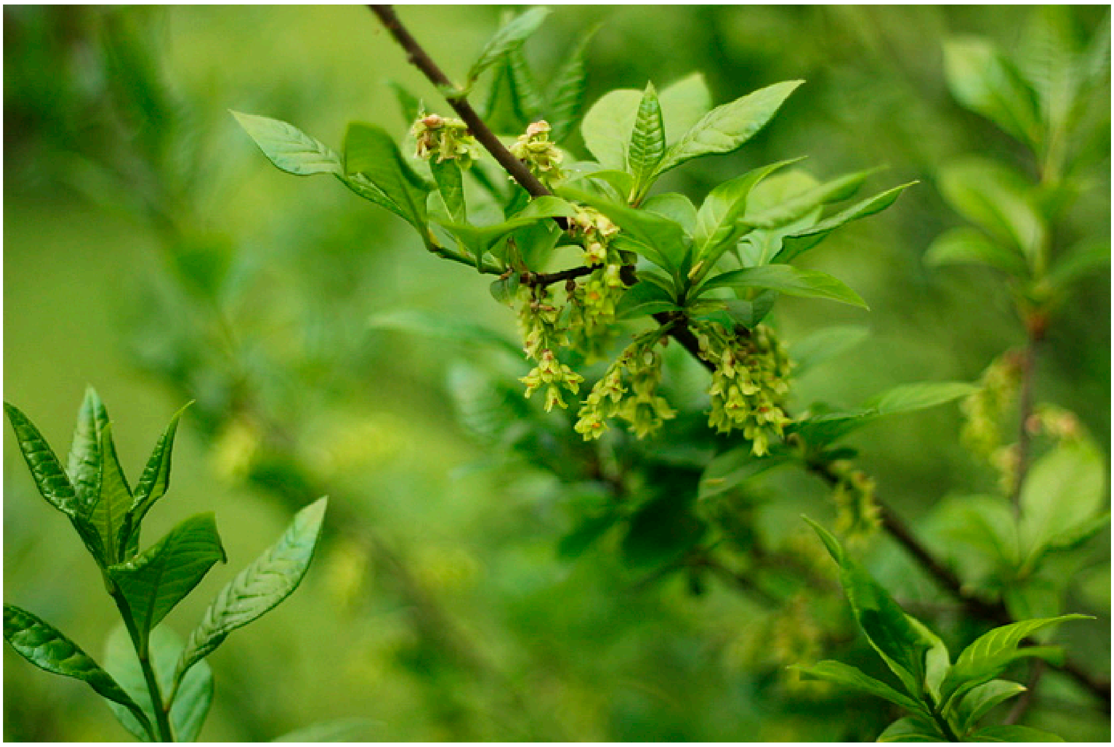

Figure S1. Picture of *Orixa japonica*.

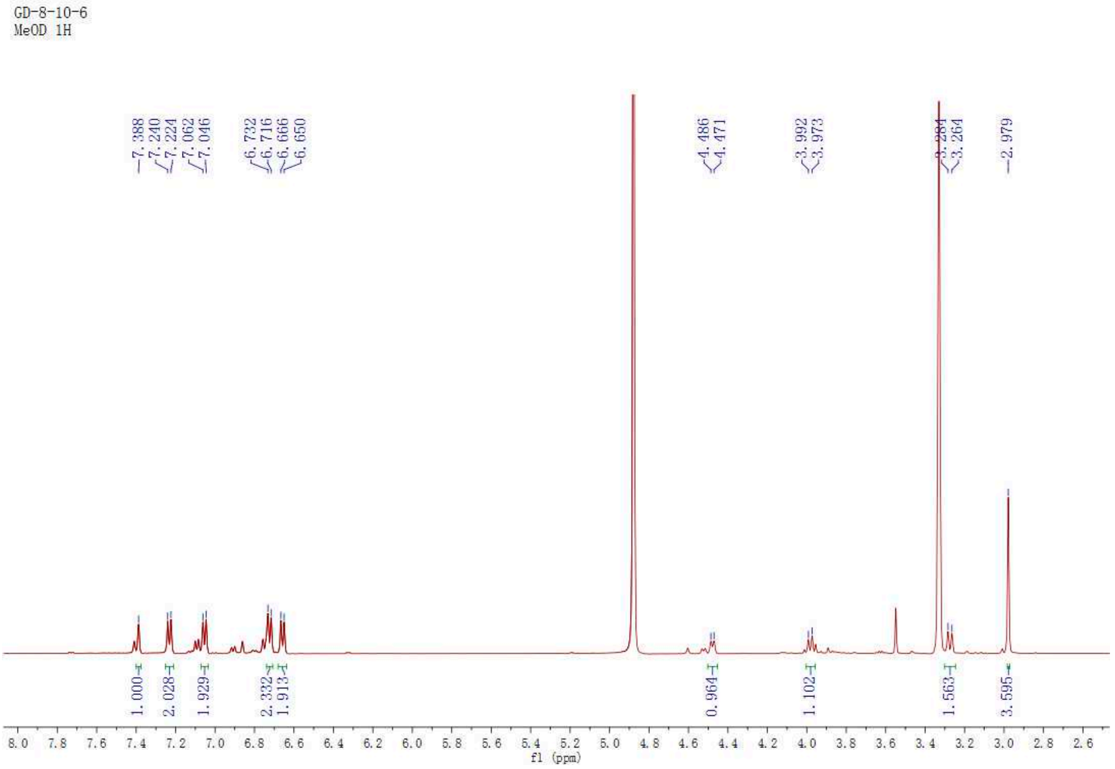

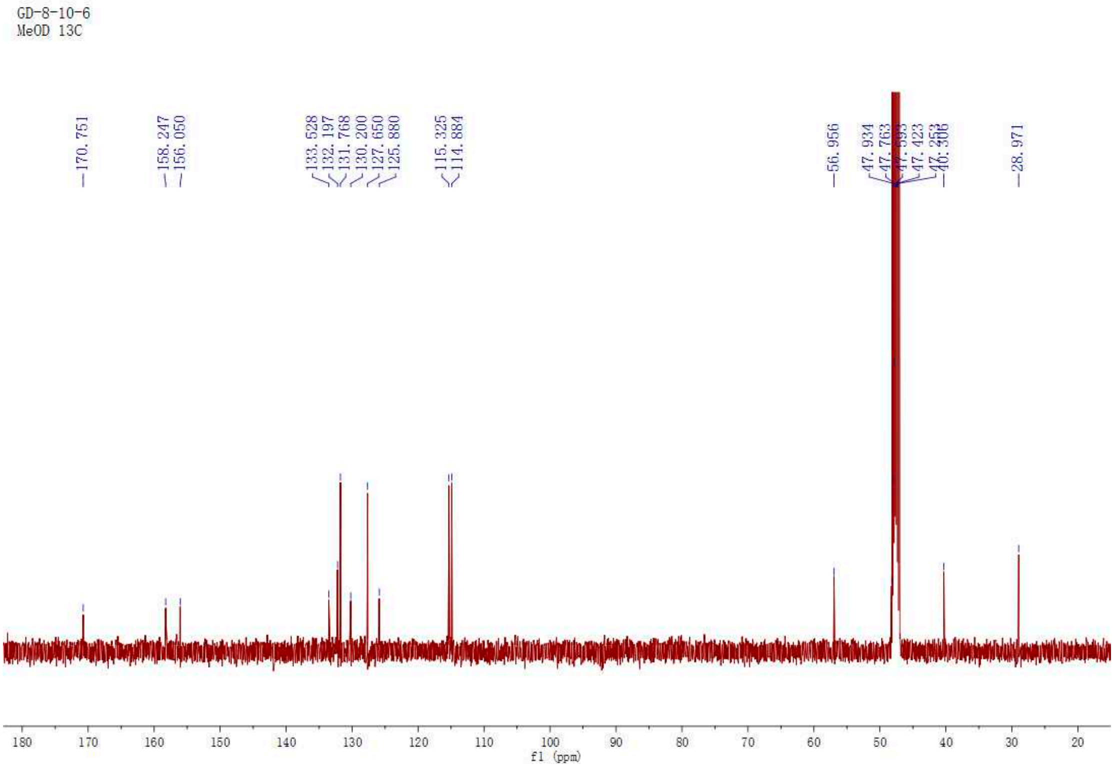

Figure S2. ^{1}H- and ^{13}C-NMR spectra of (*Z*)-3-(4-hydroxybenzylidene)-4-(4-hydroxyphenyl)-1-methylpyrrolidin-2-one.

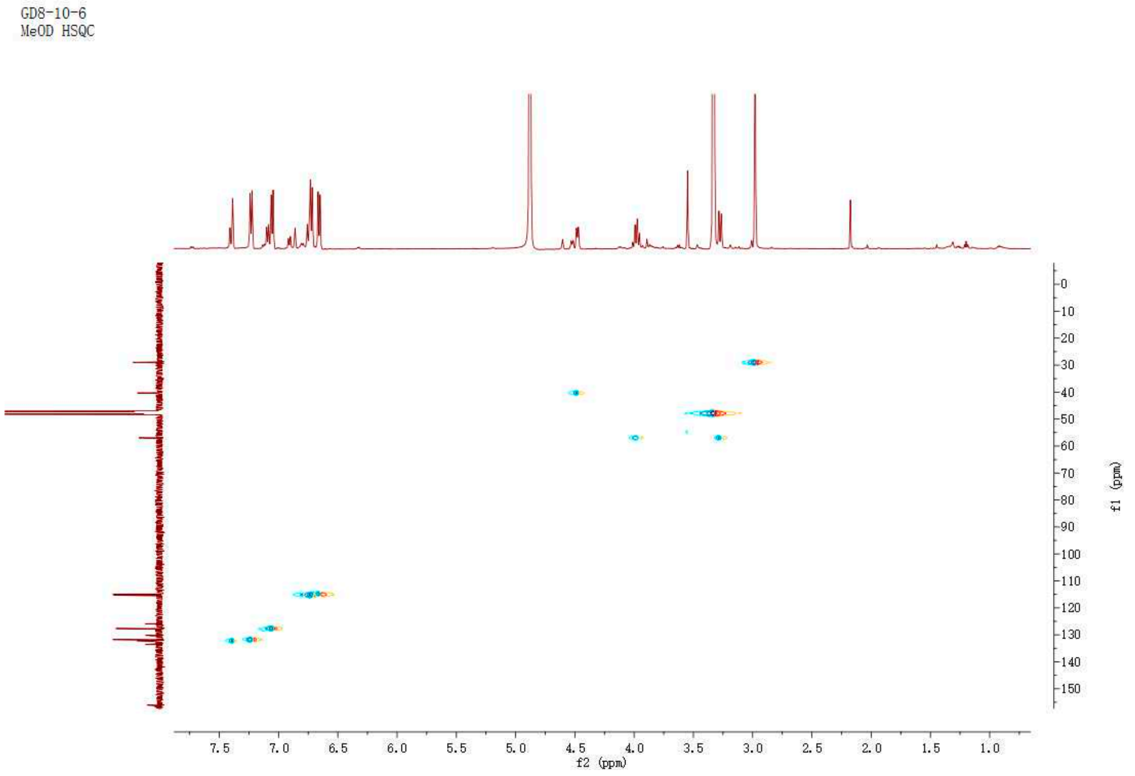

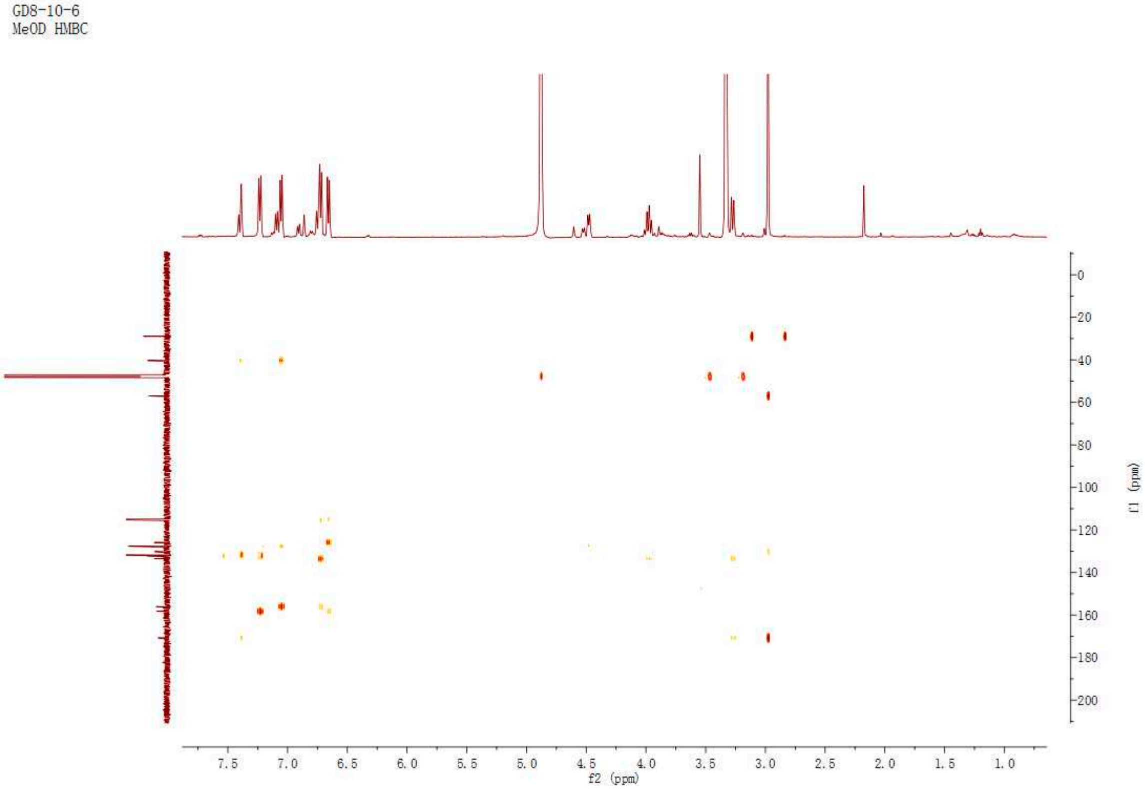

Figure S3. HSQC- and HMBC-NMR spectra of (*Z*)-3-(4-hydroxybenzylidene)-4-(4-hydroxyphenyl)-1-methylpyrrolidin-2-one.

ESI(N),8-10-6,20151225

Analysis Info

Analysis Name D:\Data\ESI\2015\2015-12\1225\8-10-6_000001.d

Acquisition Date 12/25/2015 10:22:23 AM

Sample Name 8-10-6

Instrument solariX

Acquisition Parameter

Acquisition Mode	Single MS	Acquired Scans	10	Calibration Date	Tue Nov 3 11:14:33 2015
Polarity	Negative	No. of Cell Fills	1	Data Acquisition Size	1048576
Broadband Low Mass	57.7 m/z	Source Accumulation	0.001 sec	Data Processing Size	2097152
Broadband High Mass	1000.0 m/z	Ion Accumulation Time	0.100 sec	Apodization	Sine-Bell Multiplication

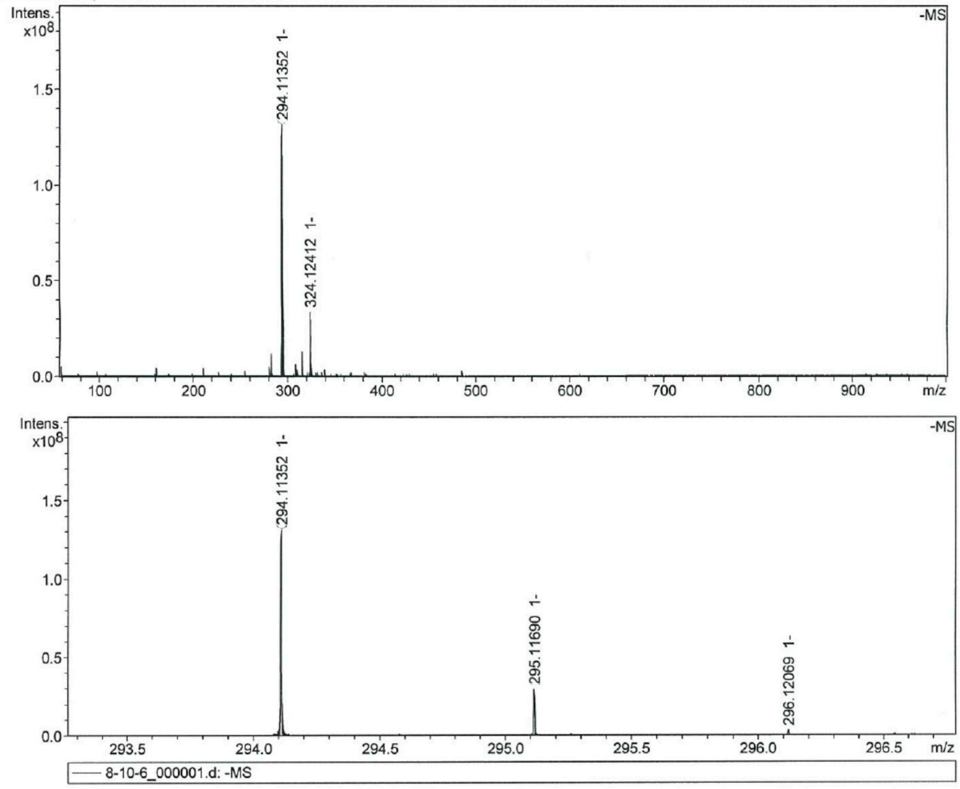

Meas. m/z	#	Ion Formula	Score	m/z	err [ppm]	Mean err [ppm]	mSigma	rdb	e⁻ Conf	N-Rule
294.113522	1	C18H16NO3	100.00	294.113567	0.2	0.0	13.6	11.5	even	ok

Figure S4. HRESIMS spectra of (*Z*)-3-(4-hydroxybenzylidene)-4-(4-hydroxyphenyl)-1-methylpyrrolidin-2-one.
